# Identifying distinct latent classes of pitch-shift response consistency: Evidence from manipulating the predictability of shift direction

**DOI:** 10.3389/fpsyg.2022.1058080

**Published:** 2022-12-14

**Authors:** Li-Hsin Ning

**Affiliations:** Department of English, National Taiwan Normal University, Taipei City, Taiwan

**Keywords:** auditory perturbation, predictability, switchers, opposers, followers

## Abstract

Auditory feedback plays an important role in regulating our vocal pitch. When pitch shifts suddenly appear in auditory feedback, the majority of the responses are opposing, correcting for the mismatch between perceived pitch and actual pitch. However, research has indicated that following responses to auditory perturbation could be common. This study attempts to explore the ways individual speakers would respond to pitch perturbation (using an opposing response or a following response) from trial to trial. Thirty-six native speakers of Mandarin produced the vowel /a/ while receiving perturbed pitch at a random time (500 ~ 700 ms) after vocal onset for a duration of 200 ms. Three blocks of 30 trials that differed in the pitch-shift stimulus direction were recorded in a randomized order: (a) the down-only condition where pitch was shifted downwards 250 cents; (b) the up-only condition where pitch was shifted upwards 250 cents; and (c) the random condition where downshifts and upshifts occurred randomly and were equally likely. The participants were instructed to ignore the pitch shifts. Results from the latent class analysis show that at the individual level across trials, 57% of participants were switchers, 28% were opposers, and 15% were followers. Our results support that speakers produce a mix of opposing and following responses when they respond to perturbed pitch. Specifically, the proportion of followers was conditional on the expectancy of pitch-shift stimulus direction: More followers were observed when the pitch-shift stimulus direction was predictable. Closer inspection of the levels of response consistency in different time phases shows that a particular mechanism (opposing or following) was initially implemented; the two mechanisms may alternate in the middle phase; and then finally, the pitch-shift response was featured as a particular mechanism near the end phase.

## Introduction

Neurocomputational models of speech-motor control such as the Directions into Velocities of Articulators (Guenther, 1995; Guenther et al., 1998; Tourville and Guenther, 2011) and the State Feedback Control (Houde and Nagarajan, 2011; Houde et al., 2013; Houde and Chang, 2015) have postulated that speech production is monitored *via feedback* and *feedforward* control mechanisms. *Feedback controllers* utilize auditory or somatosensory feedback of one’s current state to adjust motor commands. *Feedforward controllers* guide speech production by extracting previously learned motor commands. Our brains make a prediction of the sensory consequences of the issued motor actions based on an efference copy. When a mismatch occurs between predicted and actual sensory outputs in the feedback control system, corrective motion is formed to reduce the prediction error. For instance, when you speak in a noisy environment, the background noise deteriorates the audibility of your own speech from auditory feedback. In other words, the expected voice loudness does not match the perceived voice loudness. To increase the audibility of your own voice, you would involuntarily increase the voice volume (i.e., the correction motion). The phenomenon is also called the Lombard effect (Lane and Tranel, 1971). Under sustained feedback perturbations (i.e., continuous sensory errors), the feedforward system can be updated or adapted, exhibiting the plasticity of our speech motor control (Houde and Jordan, 1998, 2002).

In the past two decades, numerous studies have elucidated the role of auditory feedback in controlling voice fundamental frequency (*f*_0_) using an altered feedback paradigm (Burnett et al., 1998; Larson et al., 2001; Jones and Munhall, 2002; Natke et al., 2003; Xu et al., 2004; Zarate and Zatorre, 2005; Liu and Larson, 2007; Jones and Keough, 2008; Liu P. et al., 2010; Liu H. et al., 2010; Liu et al., 2012, 2015; Ning et al., 2014; Kim and Larson, 2019). These studies showed speakers typically produced an “opposing” response (i.e., a compensatory response that changed in the opposite direction to the pitch-shift stimulus) when they received an unexpected change in pitch through auditory feedback during vocalization. The opposing response is considered an automatic reflex whose function is to correct for the mismatch between expected pitch and heard pitch. The compensation is incomplete and partial to the shift (Larson, 1998; Liu and Larson, 2007; Sober and Brainard, 2012), generally less than 60 cents (see review in Coughler et al. (2022)), because the corrective function aims to remedy small errors rather than large ones. Speakers may also produce a “following” response that changes in the same direction as the pitch-shift stimulus. It has been widely claimed that the vast majority of pitch-shift responses are opposing, whereas following responses are less frequent. The compensatory mechanism suggests that a negative feedback loop is involved in speech production that stabilizes vocal pitch.

However, the claim that opposing responses are more prominent than following responses could be biased because the response types were classified at the *participant* level by examining a subject’s *averaged* pitch contour under a condition (Burnett et al., 1998; Hain et al., 2000; Bauer and Larson, 2003; Chen et al., 2007). More recently, several studies using a presorting technique to classify response type at the *trial* level have found that all participants produced a mix of opposing and following responses (Behroozmand et al., 2012; Korzyukov et al., 2012; Li et al., 2013; Ning, 2022). Furthermore, the following response comprised a nonnegligible proportion of pitch-shift responses: 48 ~ 56% (Behroozmand et al., 2012), 45 ~ 51% (Li et al., 2013), 45% (Korzyukov et al., 2012), 35 ~ 50% (Ning, 2022), and 10 ~ 50% (Franken et al., 2018). The generation of following responses has a few hypotheses. First, following responses could be elicited when perturbed feedback is considered an external reference (i.e., someone else’s voice), whereas opposing responses are produced when perturbed feedback is perceived as an internal reference (i.e., one’s voice; Hain et al. (2000)). Second, following responses are more likely to be triggered when participants receive larger perturbations (> 50 cents), whereas opposing responses are recruited for smaller perturbations (< 50 cents; Burnett et al. (1998)). Third, following responses could be generated when perturbation direction is misperceived (Larson et al., 2007).

Identifying a reference point (internal vs. external) does not guarantee response consistency because a speaker may demonstrate both opposing and following responses across trials or across experiments. In a pitch-shift adaptation study, Alemi et al. (2020) found that 41% of participants showed consistently opposing responses and 12% consistently exhibited following responses; however, 47% participants changed their response types from one experiment (adaptation to 100-cents pitch shift) to another (adaptation to personalized pitch shifts). The presence of a considerable proportion of following responses suggests that the two opposite mechanisms (opposing vs. following) could be activated *simultaneously* in the muscular control for vocal pitch (Li et al., 2013; Franken et al., 2018). Patel et al. (2019) proposed an expanded model of feedforward and feedback controllers for voluntary motor acts of voice *f*_0_, where speakers can *select* to operate a feedforward mode or a feedback mode. In this model, the feedforward mode is the default because it directly processes information to issue motor commands that are learned over time. In the feedforward mode, an external reference or a learned speech pattern is used as a guide for processing motor targets. In the feedback mode, auditory feedback is integrated to correct errors between the predicted pitch and actual pitch. The concept of *voluntarily* choosing either a feedforward mode or a feedback mode can be in parallel with the idea of *involuntarily* weighing between opposing (feedback mode) and following (feedforward mode) mechanisms that can be simultaneously activated for reflex-like pitch-shift responses. Ning’s (2022) investigation on tone word production further supported this weighting mechanism. The simulation results using the Bayesian approach in Ning (2022) showed that the probability of generating an opposing response was nearly the same as that of a following response when speakers were instructed to *ignore* the pitch perturbation in auditory feedback. This outcome suggests that the feedforward mode and the feedback mode should be available simultaneously. Thus, reflex-like pitch-shift responses would alternate between the two mechanisms.

Ning’s (2022) results lead to the following question: If reflex-like pitch-shift responses would alternate between opposing and following mechanisms, then in what way do they alternate over time (across trials)? More specifically, we would like to explore whether a particular mechanism (opposing or following) is initially implemented but then the two mechanisms alternate near the end of an experiment, or whether the two mechanisms alternate at the beginning but then the pitch-shift response is finalized with a particular type near the end of an experiment. Ning’s (2022) simulation results on a mix of opposing and following responses were obtained at the *population* level (i.e., pooling all participants together) and the grand average level (i.e., calculating the mean percentages). Alemi et al.’s (2020) response inconsistency was discovered across experiments. In the present study, we would like to go into further detail on individual behaviors across trials within an experiment. In the past, a great number of studies in perturbation and adaptation have indicated that the degree of vocal compensation is modulated in the ramp phase where shift magnitudes are increasingly added or dropped, and that aftereffect happens when auditory feedback returns to normal (Houde and Jordan, 1998, 2002; Max et al., 2003; Villacorta et al., 2007; Keough et al., 2013; Feng et al., 2017). We assume that plasticity across time course is involved in pitch-shift responses. Therefore, in our first research goal we aim to enumerate and identify the underlying classes (also called the latent classes) of response patterns at the *individual* level in terms of their pitch-shift response changes over time in an experiment. Characterized by conditional probabilities, data points assigned to a latent class share a common relationship. Therefore, the identified latent classes can explain the association among the observed response types across the course of time.

On the other hand, research has argued that predictability of pitch-shift stimulus direction influences the proportion of opposing and following responses. Korzyukov et al. (2012) and Behroozmand et al. (2012) each found more following responses than opposing responses occurred in the predictable condition, whereas more opposing responses than following responses were observed when downward shifts and upward shifts randomly appeared. In the present study, our second research goal is to explore whether we can predict the respondents’ class membership conditional on the predictability of pitch-shift stimulus direction. We hypothesized that if predictability plays a role, then “followers” would be more likely to appear in the predictable condition than they would be in the random condition. Finally, after identifying the latent classes of our respondents in the context of pitch perturbation, we will analyze participants’ *f*_0_ response records to estimate whether the latent classes behave differently in their acoustic profiles, including response onset time, response peak time, and absolute response peak amplitude.

## Materials and methods

### Participants

Thirty-six native speakers of Mandarin (age: 20–29 years, *M* = 23, *SD* = 2.4; 18 females) participated the research. A half of the participants learned to play musical instruments before age 12. All of them did not receive formal musical instruction or play music as an amateur in the past 5 years. None of the participants had a history of hearing or language disorders. A hearing screening test using an MAICO pure-tone audiometer (model MA 25) was administered prior to the experiment. All participants passed the hearing screening test at 20 dB bilaterally at 250, 500, 750, 1,000, 2000, 3,000, and 4,000 Hz. The participants signed informed consent approved by the institutional review board (Research Ethics Office) at National Taiwan Normal University and they were monetarily compensated for their participation.

### Procedure

A trial began with a beep sound that prompted participants to vocalize /a/ for 3 s. They were told that their voices would sound different from what they expect when they vocalized. The pitch-shift stimuli were fixed at ±250 cents. We noticed that pitch-shift responses remain consistent in response to smaller perturbations (< 250 cents), while larger perturbations (> 300 cents) result in decreased pitch-shift magnitudes (Scheerer et al., 2013). Another reason for using 250 cents was to compare the results with Ning (2022), where 250 cents triggered a phonemic shift in the lexical tone category. Although we did not test tone words in the current study, it would be interesting to discover whether the same amount of pitch-shift magnitude applied to a plain vowel would also give rise to a considerable proportion of following responses.

Participants’ voice pitch changed in three ways, corresponding to the three conditions in this study. In the down-only condition, participants’ voice pitch was shifted 250 cents down. In the up-only condition, participants’ voice pitch was shifted 250 cents up. In the random condition, participants’ voice pitches were shifted either 250 cents up or 250 cents down (randomly and equally likely). Thus, in the down-only and up-only conditions, participants were informed of the pitch-shift stimulus direction before the start of each condition so that the direction of pitch perturbation in auditory feedback was predictable, whereas in the random condition, participants could not predict the direction of pitch-shift stimuli. Participants were instructed to *ignore* the change in pitch and try to hold a steady pitch until the end of the trial.

In each trial, one pitch shift in auditory feedback (+250 cents or − 250 cents) was presented at a random time (500 to 700 ms) after vocal onset for a duration of 200 ms. The intertrial delay was 1,000 ms. In each condition, participants received a block of 30 trials. Thus, in total, they produced 90 vocalizations (30 trials × 3 conditions). To avoid the carryover effect, the order of the three conditions was counterbalanced: (a) down-only, up-only, and random; (b) random, down-only, and up-only; and (c) up-only, random, and down-only. Participants were randomly assigned to one type of order (a, b, or c). Each order had an equal number of participants. The entire experiment took approximately 30 min, including the introduction and the breaks.

Notice that we did not include control trials (normal feedback) in the experiment. The reason for this exclusion is that we did not aim to examine whether participants would respond to auditory perturbation (comparing pitch-shift trials with control trials). Numerous studies reviewed in the introduction have shown that pitch-shift responses are automatic and reflex-like responses. As our goal is to explore how the predictability of pitch-shift direction affects the proportion of opposing and following responses, all the trials involved pitch-shift stimuli.

### Apparatus

The recording was completed with participants seated in a soundproof booth. A standalone microphone (Audio Tech ATR20) was placed 1 inch away from the front of the mouth to record the voice when participants vocalized the /a/ vowel. Their voice signals were pitch-shifted through an Eventide Ultra-Harmonizer (model H7600) controlled by Max/MSP (v.8, Cycling 74) software. The pitch-shifted signals were played back in real time through AKG K240 headphones. The approximate delay of Eventide was 9 ms. To mask bone-conducted feedback signals, their speech was amplified with a 10-dB gain in the feedback channel relative to vocal output using a McLelland MAR-16P headphone amplifier. The transistor-transistor logic (TTL) pulses generated by Max/MSP (v.8, Cycling 74) to indicate the trial events and pitch-shift events (down-shifts or up-shifts) were digitized along with the participants’ vocalizations and perturbed feedback signals, using the Behringer audio interface (FCA 610). All the digitized signals were recorded using a WinDaq DI-720 acquisition device and WinDaq Pro software at a sampling rate of 8 kHz per channel.

### Data Preprocessing

The raw acoustic signals and TTL pulses in WinDaq were imported into MATALB (R2020a). The signals were split into individual trials using the TTL pulses that indicated the onset of the beep sound. For each individual trial, a 1.1-s window was chosen for pitch analysis, including a 100-ms preshift baseline, a 200-ms shift period, and an 800-ms postshift period. The segmented trials (each 1.1-s long) were converted into sound files and were fed into PRAAT for pitch estimation sampled every 10 ms. The *f*_0_ records were imported back to MATLAB and transformed into cents using the formula [cents = 1200*log2(*f*_0_/baseline)], where the baseline indicates the mean *f*_0_ of the preshift period.

Before the classification of response type, the direction of pitch-shift stimulus for each trial in the random condition was identified by the presence of the corresponding TTL pulse. Then, each vocalization (1.1-s long) was classified as an “opposing” response if the *f*_0_ contour changed in an opposite direction to the pitch-shift stimulus and the points of maximum *f*_0_ exceeded 2 standard deviations of the preshift mean, as a “following” response if the *f*_0_ contour followed the pitch-shift stimulus direction and the points of maximum *f*_0_ exceeded 2 standard deviations of the preshift mean, as a “nonresponse” if the *f*_0_ contour did not show a clear upward or downward trend and the points of maximum *f*_0_ did not exceeded 2 standard deviations of the preshift mean, and as an “error” if the *f*_0_ contour was erroneously estimated by PRAAT. The trial number (from 1 to 30) was also tagged for each vocalization.

After the classification, three measures were calculated at the trial level: the *response onset time*, the *response peak time*, and the absolute *response peak amplitude*. The response onset time was defined as the time point at which the *f*_0_ exceeded 2 standard deviations of the preshift mean and retained significance for 50 ms. The response peak time and peak amplitude were measured as the first greatest absolute pitch following the response onset. To examine the stimulus direction effect, absolute peak amplitudes were used in the data analysis.

### Statistical analyses

The first goal of the present study is to identify the underlying patterns of pitch-shift response changes over time (i.e., in a sequence of 30 trials). Latent class analysis was used because it can probabilistically group each observation into a latent class based on the manifest variables. Our observed manifest variables were the response types in the 30 trials (i.e., opposing, following, nonresponse, or error), which were nominal and were assumed to be locally independent. The poLCA() function in the poLCA package (Linzer and Lewis, 2011) estimates the latent class model by maximizing the following log-likelihood function using the Expectation–Maximization (EM) algorithm:


$$\ln L=\overset{N}{\underset{i=1}{\sum }}\ln\overset{R}{\underset{r=1}{\sum }}p_{r}\overset{J}{\underset{j=1}{\prod }}\overset{K_{j}}{\underset{k=1}{\prod }}{\left(\pi_{jrk}\right)}^{Y_{ijk}}$$

where we observe *J* polytomous manifest variables (*J* = 30 in our study), each of which contains $K_{j}$ possible outcomes (*K* = 4), for individuals *i* = 1, …, *N*. In the formula, *π_jrk_* denotes the class-conditional probability that an observation in class *r* = 1, …, *R* produces the *k*th outcome on the *j*th variable. The values of $p_{r}$ indicate the prior probabilities of latent class membership. One of the benefits of using latent class analysis is that it provides a number of fit indices available for model selection and for choosing an appropriate number of latent classes. The fit indices include Akaike information criterion (AIC), Bayesian information criterion (BIC), and entropy (a measure of dispersion). Preferred models are those that minimize values of these fit indices, except for the entropy.

Our second research question aims to explore whether the class membership of the respondents would depend on the predictability of pitch-shift stimulus direction (down-only, up-only, or random). To address this question, latent class regression modeling, which enables the inclusion of covariates to predict individuals’ latent class membership, was used. It allows individuals’ priors to vary depending on the observed covariate (pitch-shift stimulus direction) and it estimates the coefficients of the covariate simultaneously as part of the latent class model. Both the latent class analysis and the latent class regression modeling were performed in R Core Team (2021) using the poLCA package (Linzer and Lewis, 2011).

Finally, we investigated whether significant differences occurred between the identified classes concerning the response onset time, the response peak time, and the response peak amplitude. Linear mixed effects models, which included random effects associated with individual participants, were conducted in R Core Team (2021) using the afex (Singmann et al., 2021) and emmeans (Lenth, 2020) packages. The within-subject fixed effects included (pitch-shift) stimulus direction (down-only, up-only, or random), and response type (opposing or following). The between-subject fixed effect was the latent class predicted from the latent class regression models. To handle violations of sphericity, the degrees of freedom were Greenhouse–Geisser corrected. For *post hoc* simultaneous comparisons, the *p* values were adjusted using the Tukey’s honestly significant difference correction (with α set at 0.05).

## Results

### Identifying the class membership

**Figure 1:**
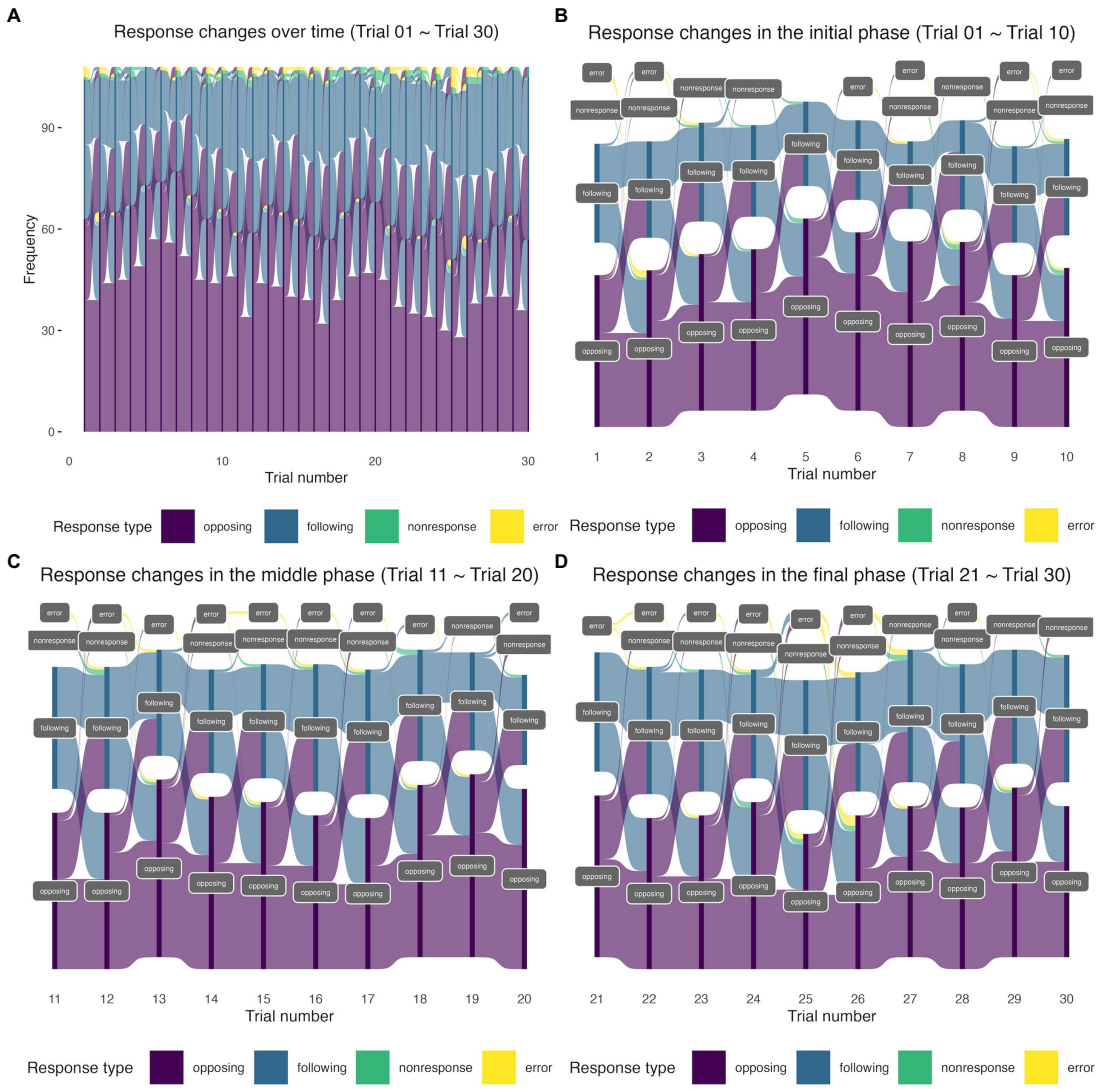
**(A)** The alluvial diagram of response type counts in the 30 trials. **(B)** The Sankey diagram of response type counts in the initial phase (the first 10 trials). **(C)** The Sankey diagram of response type counts in the middle phase (the middle 10 trials). **(D)** The Sankey diagram of response type counts in the final phase (the last 10 trials). The height of the nodes (dark purple, dark blue, dark green, and dark yellow) shows the frequency (counts) of each response type (coded in different colors) in each trial. The curves between the trials coded in light purple, light blue, light green, and light yellow represent the transitions from one response type to another. The widths of the curves are proportional to the transition rate. Sankey plots are similar to alluvial plots but differ in the presence of spaces between nodes at each stage (i.e., trial, in our case). The presence of spaces between nodes at one trial in the Sankey plots enhances the visualization of the transition flow.

The distributions of the possible response outcomes over time are presented in Table 1 and Figure 1. The alluvial and Sankey diagrams in Figure 1 show the four possible response types (opposing, following, nonresponse, and error) observed in the 30 trials. The same visualization method has been used to demonstrate the interview mode switches in an annual longitudinal Understanding Society survey (Cernat and Sakshaug, 2021). It is evident from Figure 1 that a large proportion of “switchers” switched between opposing responses and following responses over time (the transition flow between purple and blue nodes in the middle of the diagrams). In addition, a large proportion of “opposers” tended to remain the same (the dark purple nodes at the bottom of the diagram). We also observed a considerable proportion of “followers” (the dark blue nodes at the top of the diagram). The nonresponse and error types were infrequently seen (1% in Table 1). Notice that latent classes refer to the underlying structures that can be derived from the data. They may or may not correspond to a particular observed outcome (in our case, a particular response type). Latent classes may also represent a relationship (or a combination) of several outcome variables (such as our switcher class). The distinct names we use here (switchers, opposers, and followers) are descriptive of the behavioral patterns of our respondents but do not imply that participants voluntarily choose to operate as one of them. The latent classes that were temporarily named here (switchers, opposers, and followers) have to be justified *via* statistical modeling.

**Table 1:** The frequency (counts) of response types in the 30 trials on the sample size of 36 participants in three conditions.

	**Down-only condition**				**Up-only condition**				**Random condition**				**Total (the sum of all three conditions)**			
	**Opposing**	**Following**	**Nonresponse**	**Error**	**Opposing**	**Following**	**Nonresponse**	**Error**	**Opposing**	**Following**	**Nonresponse**	**Error**	**Opposing**	**Following**	**Nonresponse**	**Error**
**Trial 01**	19	15	0	2	22	14	0	0	22	12	1	1	63	41	1	3
**Trial 02**	24	11	0	1	19	15	1	1	22	14	0	0	65	40	1	2
**Trial 03**	22	14	0	0	25	11	0	0	18	16	2	0	65	41	2	0
**Trial 04**	24	11	1	0	24	12	0	0	19	15	2	0	67	38	3	0
**Trial 05**	19	17	0	0	26	10	0	0	28	8	0	0	73	35	0	0
**Trial 06**	25	11	0	0	26	9	0	1	23	13	0	0	74	33	0	1
**Trial 07**	22	13	1	0	28	6	1	1	27	9	0	0	77	28	2	1
**Trial 08**	24	12	0	0	23	12	1	0	23	13	0	0	70	37	1	0
**Trial 09**	19	17	0	0	19	15	1	1	25	8	2	1	63	40	3	2
**Trial 10**	22	13	1	0	23	12	0	1	21	15	0	0	66	40	1	1
**Trial 11**	16	19	0	1	24	10	2	0	19	17	0	0	59	46	2	1
**Trial 12**	15	21	0	0	20	13	1	2	24	12	0	0	59	46	1	2
**Trial 13**	21	15	0	0	26	10	0	0	20	15	0	1	67	40	0	1
**Trial 14**	20	14	1	1	21	15	0	0	24	10	1	1	65	39	2	2
**Trial 15**	22	14	0	0	21	14	1	0	20	15	0	1	63	43	1	1
**Trial 16**	16	20	0	0	21	13	1	1	21	15	0	0	58	48	1	1
**Trial 17**	14	21	1	0	18	16	1	1	25	10	1	0	57	47	3	1
**Trial 18**	18	17	0	1	24	12	0	0	23	13	0	0	65	42	0	1
**Trial 19**	19	17	0	0	24	11	1	0	26	9	1	0	69	37	2	0
**Trial 20**	22	13	1	0	25	8	3	0	21	13	1	1	68	34	5	1
**Trial 21**	15	19	0	2	25	11	0	0	21	15	0	0	61	45	0	2
**Trial 22**	13	21	1	1	23	12	1	0	21	15	0	0	57	48	2	1
**Trial 23**	17	17	2	0	19	16	1	0	22	13	0	1	58	46	3	1
**Trial 24**	16	19	0	1	24	11	1	0	23	11	1	1	63	41	2	2
**Trial 25**	19	15	1	1	15	18	0	3	17	16	1	2	51	49	2	6
**Trial 26**	16	17	1	2	23	13	0	0	19	15	1	1	58	45	2	3
**Trial 27**	17	19	0	0	22	14	0	0	18	17	1	0	57	50	1	0
**Trial 28**	15	20	0	1	25	11	0	0	21	14	1	0	61	45	1	1
**Trial 29**	17	19	0	0	22	13	1	0	25	11	0	0	64	43	1	0
**Trial 30**	15	21	0	0	24	10	2	0	18	17	1	0	57	48	3	0
**Proportion**	52%	46%	1%	1%	63%	34%	2%	1%	61%	37%	1%	1%	59%	39%	1%	1%

To estimate an appropriate number of latent classes, the response types over the 30 trials were used as dependent variables in the latent class analyses. We started from a simpler model with only two classes (nclass = 2), and transitioned to a more complex model with five classes (nclass = 5). Because the EM algorithm depends on the initial parameter values selected in the first iteration, it may find a local maximum rather than a global maximum for a parameter. To avoid this problem, we estimated the latent class model 30 times (nrep = 30) using different initial parameter values. Their fit indices are presented in Table 2. The AIC and BIC values pointed towards the two-class model as the best fitting one, whereas entropy did not provide any suggestion. However, a closer inspection of the AIC differences shows the three-class model was only 2 units larger than the two-class model was ( $\Delta =AIC_{3}-AIC_{2}$ and 2<Δ<4 ), indicating strong support for the more complicated three-class model (Burnham and Anderson, 2004). Additionally, the visual representation of the response outcomes displayed in Figure 1 suggests three subgroups existed. Therefore, we opted for the three-class solution.

**Table 2:** The fit indices of latent class analyses with two to five classes.

**Number of classes**	**LL**	**Parameters**	***n***	**AIC**	**BIC**	**Entropy**
2	−2,388	155	108	5,087	5,502	4.68
3	−2,312	233	108	5,089	5,715	4.68
4	−2,299	311	108	5,220	6,054	4.68
5	−2,245	389	108	5,267	6,310	4.68

LL, the maximum value of the estimated model log-likelihood; parameters, the number of estimated parameters; *n*, the number of fully observed cases; AIC, Akaike information criterion; BIC, Bayesian information criterion; Entropy, the measure of dispersion in the probability mass function.

**Figure 2:**
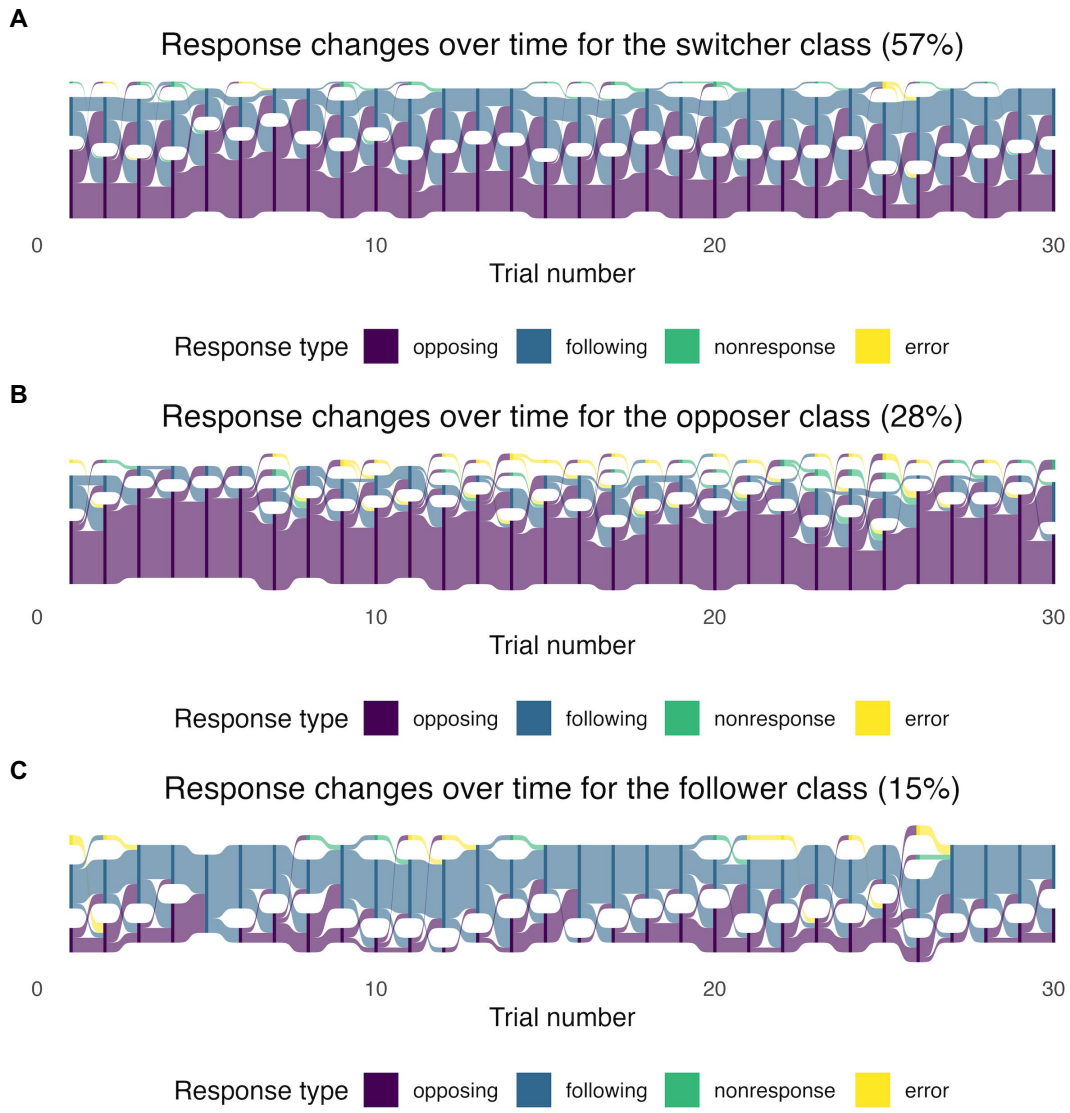
The Sankey diagrams of response-type proportions in the 30 trials based on the latent class membership: switchers **(A)**, opposers **(B)**, and followers **(C)**. The height of the nodes (dark purple, dark blue, dark green, and dark yellow) shows the frequency (counts) of each response type (coded in different colors) in each trial. The curves between the trials coded in light purple, light blue, light green, and light yellow represent the transitions from one response type to another. The width of the curves is proportional to the transition rate.

In the three-class model, each observation was assigned a latent class based on the class with the highest likelihood. Then, we separated the observations by each latent class and recreated the Sankey diagrams in Figure 2. The largest class, which we called “switchers,” included 57% of the sample where opposing and following responses alternated over time. The transition can be visualized from the wide light purple and light blue curves across the trials in Figure 2A. The second class was called “opposers” and it included 28% of the sample. The respondents in the second class tended to produce consistently opposing responses (Figure 2B). The third class was called “followers,” which included respondents who mainly used following responses in the pitch-shift task. The followers occupied around 15% of the sample (Figure 2C).

### Estimating class membership conditional on the predictability of stimulus direction

**Figure 3:**
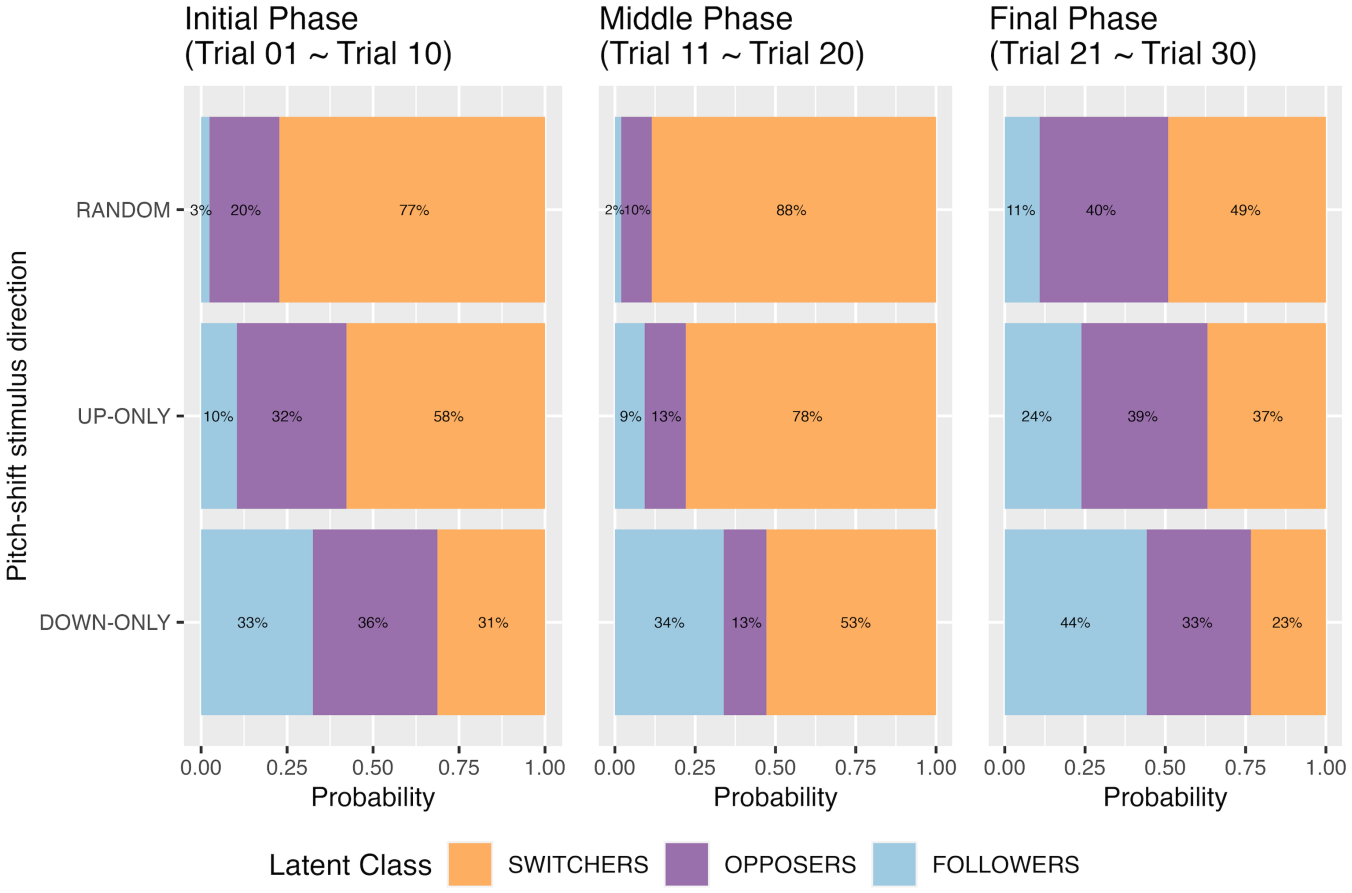
Predicted prior probability of latent class membership for the three pitch-shift stimulus directions at the initial phase (the first 10 trials), the middle phase (the middle 10 trials), and the final phase (the last 10 trials). Results are from the three-class latent class regression models.

In the previous section, we identified three latent classes of respondents: switchers, opposers, and followers. We may expect that falling into one of these three classes is a function of the predictability of the pitch-shift stimulus direction, because Korzyukov et al. (2012) argued that predictable pitch perturbations lead to a reduced proportion of opposing responses and an increasing number of following responses. To investigate the hypothesis, three-class latent class regression modeling was performed with pitch-shift stimulus direction as the covariate variable. However, fitting 30 trials altogether in a three-class latent class regression model produced negative degrees of freedom, showing that the model tried to estimate more parameters than it was possible to estimate. Thus, we ran the three-class latent class regression models separately for the first 10 trials (initial phase), the middle 10 trials (middle phase), and the last 10 trials (final phase). The model results are summarized in Table 3. Visual representations depicting the predicted prior probabilities of latent class membership conditional on pitch-shift stimulus direction are displayed in Figure 3.

**Table 3:** Results from the three-class latent class regression models using pitch-shift stimulus direction as a covariate variable.

		**Opposers**				**Followers**			
		**Est.**	**SE**	***t***	***p***	**Est.**	**SE**	***t***	***p***
**Initial phase: Trial 01 ~ 10**	**Intercept**	0.89	0.98	0.91	0.37	1.79	1.30	1.37	0.18
	**DIRECTION**	−0.74	0.48	−1.55	0.13	−1.75	0.87	−2.00	0.04*
**Middle phase:** **Trial 11 ~ 20**	**Intercept**	−0.95	2.55	−0.37	0.71	1.24	1.49	0.83	0.41
	**DIRECTION**	−0.42	1.47	−0.30	0.77	−1.69	1.14	−1.48	0.15
**Final phase:** **Trial 21 ~ 30**	**Intercept**	0.60	1.03	0.58	0.57	1.71	0.90	1.91	0.07
	**DIRECTION**	−0.27	0.45	−0.60	0.56	−1.07	0.45	−2.37	0.03*

The switcher class was used as the reference category.

Est. = estimated coefficient; SE = standard error.

*p < 0.05.

Comparing three pitch-shift stimulus directions, we observed that when the participants responded to *unpredictable* random pitch-shifts, they were less likely to be in the “follower” class (5% by averaging the topmost three blue bars in Figure 3) but more likely to be a switcher (71% by averaging the topmost three orange bars in Figure 3). For the two *predictable* directions (up-only and down-only), the probabilities of being classified into the “follower” class were significantly higher than they were for the random condition. However, the two predictable directions did not expect the same proportion of followers. When the participants responded to predictable down-shifts, the probability of being a follower was 37% (by averaging the bottom three blue bars in Figure 3); when they responded to predictable up-shifts, the probability of being a follower was only 14% (by averaging the middle three blue bars in Figure 3). In other words, our hypothesis that followers would be more likely to appear in the predictable condition than they would in the random condition was in general supported. The results also suggest a directional difference for the predictable pitch-shifts.

The predicted proportions of the opposer class in the three time phases were not affected by pitch-shift stimulus direction, which can be seen from the insignificance in Table 3 and the roughly same-sized purple bars from top to bottom in Figure 3. However, an interesting pattern on the predicted proportions of the three classes over time was observed. Participants who were identified as an opposer in the initial phase may become switchers in the middle phase, leading to a reduction in the opposer class and an increase in the switcher class in the middle phase, compared to the initial phase. When time approached the end of the recording block (i.e., the last 10 trials), the predicted proportion of switchers reduced, accompanied by the increasing proportions of opposers and followers. It seems that a particular response type was finalized after the two mechanisms alternated in the middle phase.

### Calculating the behavioral differences among the latent classes

The third research question investigated whether significant differences exist among the latent classes conditional on stimulus direction and response type for the following three measures: response onset time, response peak time, and response peak amplitude. The latent classes were predicted from the latent class regression models presented in the previous section. Values of the three measures across latent class, stimulus direction, and response type are displayed in Table 4. Results from the linear mixed-effects models are summarized in Table 5.

**Table 4:** Average response onset time, response peak time, and response peak amplitude (SE) as a function of latent class, stimulus direction, and response type.

**Latent class**	**Stimulus direction**	**Response type**	**Response onset time (ms)**	**Response peak time (ms)**	**Response peak amplitude (cents)**
Switcher	Down-only	Opposing response	196 (22)	402 (30)	31 (3)
		Following response	229 (26)	471 (30)	36 (5)
	Up-only	Opposing response	230 (25)	440 (28)	37 (4)
		Following response	229 (27)	429 (30)	32 (3)
	Random	Opposing response	212 (22)	430 (28)	44 (7)
		Following response	227 (27)	454 (32)	35 (5)
Opposer	Down-only	Opposing response	195 (20)	372 (28)	41 (5)
		Following response	249 (30)	464 (31)	33 (3)
	Up-only	Opposing response	221 (24)	448 (29)	43 (5)
		Following response	222 (28)	448 (30)	32 (4)
	Random	Opposing response	242 (27)	446 (31)	39 (5)
		Following response	207 (26)	439 (36)	49 (8)
Follower	Down-only	Opposing response	200 (24)	401 (29)	36 (5)
		Following response	214 (26)	459 (31)	53 (9)
	Up-only	Opposing response	206 (19)	396 (23)	36 (8)
		Following response	186 (23)	350 (32)	93 (16)
	Random	Opposing response	146 (11)	336 (24)	55 (9)
		Following response	205 (25)	458 (30)	58 (11)

**Table 5:** Summary table of the linear mixed-effects models on response onset time, response peak time, and response peak amplitude.

	**Response onset time**		**Response peak time**		**Response peak amplitude**	
**Effect**	***F***	***p***	***F***	***p***	***F***	***p***
Latent class	*F*(2, 2033.40) = 2.15	0.117	*F*(2, 1947.54) = 1.03	0.358	*F*(2, 2372.86) = 5.91	0.003**
Stimulus direction	*F*(2, 133.12) = 0.26	0.768	*F*(2, 67.61) = 0.59	0.557	*F*(2, 41.37) = 4.98	0.012*
Response type	*F*(1, 68.09) = 1.51	0.224	*F*(1, 43.32) = 4.05	0.049*	*F*(1, 37.65) = 3.85	0.057
Latent class × stimulus direction	*F*(4, 1526.69) = 1.53	0.192	*F*(4, 1468.83) = 3.61	0.006**	*F*(4, 1955.20) = 2.44	0.045*
Latent class × response type	*F*(2, 1443.32) = 1.11	0.331	*F*(2, 1718.99) = 1.90	0.151	*F*(2, 1578.55) = 4.81	0.008**
Stimulus direction × response type	*F*(2, 89.34) = 2.38	0.099	*F*(2, 60.98) = 5.18	0.008**	*F*(2, 61.36) = 1.09	0.343
Latent class × stimulus direction × response type	*F*(4, 1135.93) = 1.92	0.105	*F*(4, 1313.02) = 2.26	0.061	*F*(4, 1526.65) = 5.79	<0.001***

*p < 0.50, **p < 0.01, ***p < 0.001.

#### Response onset time

Linear mixed-effects models performed on the response onset time, incorporating latent class, stimulus direction, and response type as fixed effects and treating individual participants as a random effect, revealed no significant main effects and no significant interaction effects. In general, participants responded to pitch perturbation approximately 213 ± 7 ms after pitch-shift stimulus onset, unaffected by latent class, stimulus direction, and response type.

#### Response peak time

**Figure 4:**
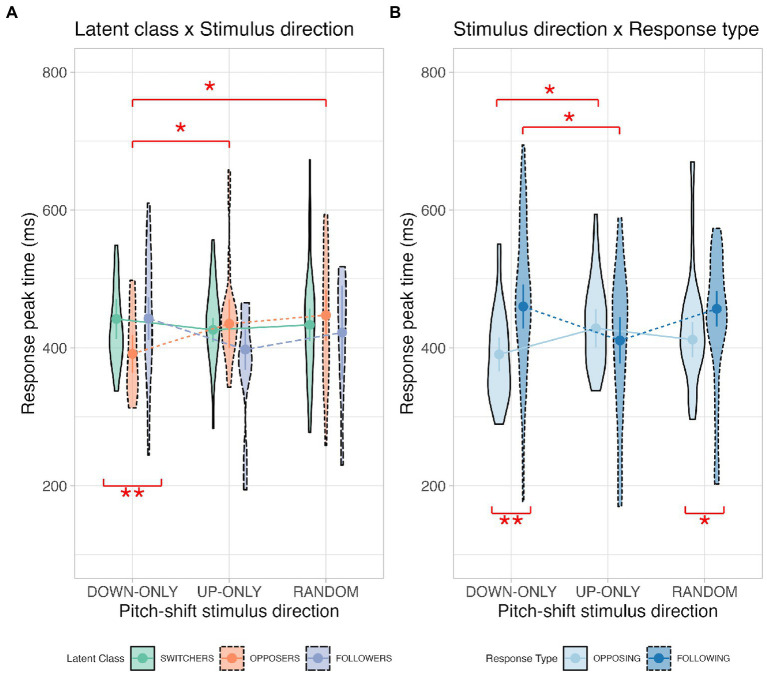
Violin plots illustrating the latent class × stimulus direction interaction (Panel **A**) and the stimulus direction × response type interaction (Panel **B**) for the response peak time. Definitions for the violin plots: Means superimposed with error bars are connected by lines, violin shapes extend to 1.5 times the interquartile range, and widths of the violin shapes represent density distributions.

As for the response peak time, results from the linear mixed-effects model show that a significant main effect of response type, *F*(1, 43.32) = 4.05, *p* = 0.049, a significant interaction between latent class and stimulus direction, *F*(4, 1468.83) = 3.61, *p* = 0.006, and a significant interaction between stimulus direction and response type, *F*(2, 60.98) = 5.18, *p* = 0.008, were found. *Post hoc* comparisons for response type indicate that opposing responses (410 ± 9 ms) had significantly faster peak times than following responses had (442 ± 14 ms). Violin plots illustrating the interaction effects for the response peak time are displayed in Figure 4.

To determine the latent class effect on response peak time under different stimulus directions (latent class × stimulus direction), we performed simple main-effects analyses for the down-only, up-only, and random conditions. The results revealed a significant latent class effect for the down-only condition, where opposers (391 ± 16 ms) had faster peak times than both switchers (442 ± 13 ms; *p* = 0.006) and followers (443 ± 13 ms; *p* = 0.010) had. Simple main-effects analyses were also conducted to examine the effect of stimulus direction on response peak time for the switcher, opposer, and follower classes. Our analyses revealed a significant stimulus direction effect for the opposer class, where the down-only condition (391 ± 16 ms) had faster peak times than both the up-only (435 ± 14 ms; *p* = 0.050) and random conditions (447 ± 17 ms; *p* = 0.019) had.

As for the interaction between stimulus direction and response type (stimulus direction × response type), we investigated the stimulus direction effect on response peak time under different response types. The results show that in opposing response, the down-only condition (391 ± 11 ms) had significantly faster peak times than the up-only condition (428 ± 13 ms; *p* = 0.043) had, whereas in following response, the up-only condition (411 ± 16 ms) had significantly faster peak times than both the down-only condition (460 ± 20 ms; *p* = 0.028) and the random condition (457 ± 18 ms; *p* = 0.027) had. Simple main-effects analyses were also performed to examine the effect of response type on response peak time for the down-only, up-only, and random conditions. The results show that opposing responses had significantly faster peak times than following responses for the down-only (opposing: 391 ± 11 ms vs. following: 460 ± 20 ms; *p* = 0.002) and random conditions (opposing: 412 ± 18 ms vs. following: 457 ± 18 ms; *p* = 0.038) had.

Overall, these findings suggest that our opposers had faster peak times than the other two classes had, particularly when the stimulus direction was downwards and predictable. Additionally, pitch-increasing responses (i.e., oppose downshifts or follow upshifts) were significantly faster peak times than pitch-decreasing responses (i.e., oppose upshifts or follow downshifts).

#### Response peak amplitude

**Figure 5:**
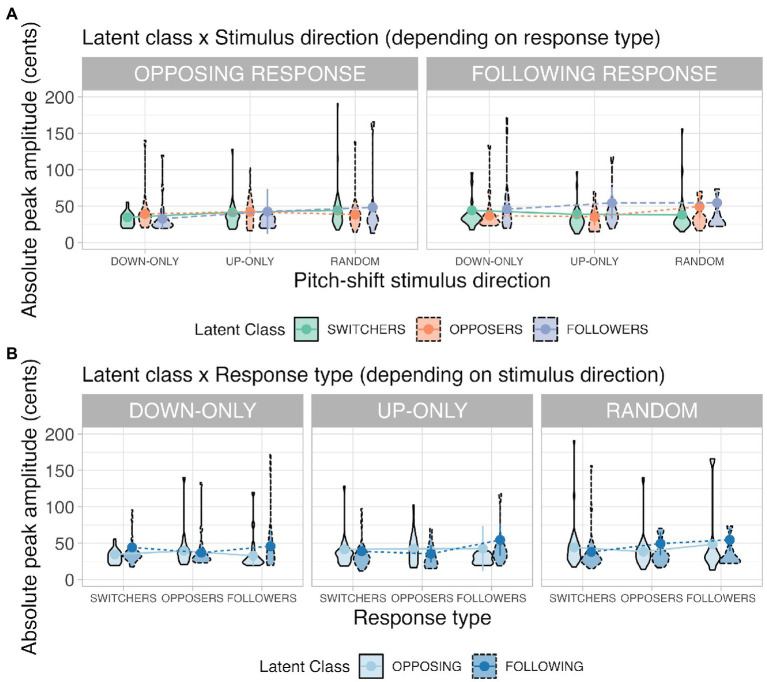
Violin plots illustrating the latent class × stimulus direction interaction (Panel **A**) and the latent class × response type interaction (Panel **B**) for the response peak amplitude. Definitions for the violin plots: Means superimposed with error bars are connected by lines, violin shapes extend to 1.5 times the interquartile range, and widths of the violin shapes represent density distributions.

Results from the linear mixed-effects model performed on the response peak amplitude show that significant main effects of latent class, *F*(2, 2372.86) = 5.91, *p* = 0.003, and stimulus direction, *F*(2, 41.37) = 4.98, *p* = 0.012, were observed. *Post hoc* comparisons for latent class indicate that switchers (40 ± 5 cents) and opposers (40 ± 5 cents) had significantly smaller peak amplitudes than followers had (46 ± 5 cents; switchers = opposers < followers). *Post hoc* comparisons for stimulus direction shows that the down-only condition (39 ± 4 cents) had significantly smaller peak amplitudes than the random condition had (46 ± 5 cents; down-only < random), whereas the up-only condition (43 ± 5 cents) fell in between (up-only = down-only; up-only = random). Significant interactions also existed between latent class and stimulus direction, *F*(4, 1955.20) = 2.44, *p* = 0.045, between latent class and response type, *F*(2, 1578.55) = 4.81, *p* = 0.008, and among the three factors, *F*(4, 1526.65) = 5.79, *p* < 0.001. Because the second-order interaction (latent class × stimulus direction × response type) was significant, it means the simple (first-order) interactions of any two factors varied with changes in a third factor. To determine how the first-order interactions work, we first conducted simple interaction effects by specifying the third factor’s level. Then we performed simple main-effects analyses for significant first-order interactions (depicted in Figure 5).

##### (a) Latent class × stimulus direction (depending on response type)

When the response type was an opposing response, a significant interaction occurred between latent class and stimulus direction, *F*(4, 1345.17) = 3.68, *p* = 0.007. Simple main-effects analyses in the random condition reveal that opposers’ opposing responses (38 ± 5 cents) had significantly smaller peak amplitudes than the switchers’ opposing responses had (44 ± 5 cents; opposers < switchers; *p* = 0.036).

When the response type was a following response, a significant interaction occurred between latent class and stimulus direction, *F*(4, 1118.11) = 4.09, *p* = 0.003. Simple main-effects analyses in the up-only condition revealed that switchers (38 ± 6 cents; *p* = 0.001) and opposers’ (36 ± 7 cents; *p* = 0.012) following responses had significantly smaller peak amplitudes than the followers’ (55 ± 7 cents) following responses had (switchers = opposers < followers). In the random condition, switchers’ (38 ± 5 cents) following responses had significantly smaller peak amplitudes than the opposers (50 ± 7 cents; *p* = 0.019) and followers’ (53 ± 7 cents; *p* = 0.023) following responses had (switchers < opposers = followers).

Results from the latent class × stimulus direction interaction indicate that in the random condition, opposers had *small* opposing responses, whereas followers had *large* following responses. The switchers overall had medium-sized pitch-shift responses (larger opposing responses than opposers had but smaller following responses than followers had).

##### (b) Latent class × response type (depending on stimulus direction)

When the stimulus direction was down-only, a significant interaction appeared between latent class and response type, *F*(2, 250.12) = 4.82, *p* = 0.009. Simple main-effects analyses show that in the switcher class, participants’ opposing responses (35 ± 4 cents) were significantly smaller than their following responses (45 ± 6 cents) were in the down-only condition (opposing < following; *p* = 0.018); in the follower class, their following responses (46 ± 10 cents) were significantly larger than their opposing responses (32 ± 4 cents) were in the down-only condition (following > opposing; *p* = 0.001).

When the stimulus direction was up-only, a significant interaction appeared between latent class and response type, *F*(2, 530.84) = 4.79, *p* = 0.009. Simple main effects analyses in the opposer class revealed that their opposing responses (44 ± 7 cents) were significantly larger than their following responses (36 ± 7 cents) were in the up-only condition (opposing > following; *p* = 0.049); in the follower class, participants’ following responses (55 ± 7 cents) were significantly larger than their opposing responses (42 ± 8 cents) were in the up-only condition (following > opposing; *p* = 0.035).

When the stimulus direction was random, a significant interaction appeared between latent class and response type, *F*(2, 1010.21) = 7.86, *p* < 0.001. Simple main effects analyses showed that in the switcher class, their opposing responses (44 ± 5cents) were significantly larger than their following responses (38 ± 6 cents) were in the random condition (opposing > following; *p* = 0.003); in the opposer class, their opposing responses (38 ± 5 cents) were significantly smaller than their following responses (48 ± 6 cents) were in the random condition (opposing < following; *p* = 0.019).

Results from the latent class × response type interaction indicate that followers’ following responses were consistently *larger* than their opposing responses were, irrespective of the stimulus direction. For switchers and followers in the down-only and up-only conditions, their pitch-increasing responses (i.e., oppose downshifts or follow upshifts) were *smaller* than their pitch-decreasing responses (i.e., oppose upshifts or follow downshifts) were. The divergence between switchers and opposers lied in the random condition. When the stimulus direction was unpredictable, switchers’ opposing responses were *larger* than their following responses were, whereas opposers’ opposing responses were *smaller* than their following responses were.

## Discussion

### Interspeaker variability: Switchers, opposers, and followers

Our first research question aimed to identify the underlying classes of speakers at the individual level across trials in an experiment. Our data showed large interspeaker variability occurred in terms of response consistency *across trials* (i.e., whether particular mechanism, opposing or following, was mainly used, or whether the two mechanisms alternated over time). The latent class analyses identified three subgroups in our respondents: 57% switchers, 28% opposers, and 15% followers. The distribution of the three subgroups, to a certain degree, resembled the distribution in Alemi et al. (2020), who found 47% switchers, 41% opposers, and 12% followers. The methodological differences between our study and Alemi et al. (2020) lie in three aspects. First, we examined vocal responses to sudden and short pitch shifts at a random point during vocalizations (compensation study), whereas Alemi et al. (2020) examined adaptive responses to sustained pitch shifts that were applied from vocal onset to the end of vocalizations (adaptation study). Second, the subgroups in our study were obtained from latent class analysis, whereas the subgroups in Alemi et al. were acquired from observing response magnitudes in the 100 trials of the hold phase (where maximum pitch shift was maintained). Third, our latent classes represented the degree of response consistency *across trials* within an experiment, whereas the subgroups in Alemi et al. represented the degree of response consistency *across experiments*. Nevertheless, despite the methodological differences, the large proportion of inconsistent responses (i.e., switchers) supports the claim that two opposite mechanisms, opposing and following, could be activated *simultaneously* in the muscular control for vocal pitch (Li et al., 2013; Franken et al., 2018).

Large interspeaker variability has been found in adaptation of speech production to sustained feedback perturbation. A tradeoff might occur between auditory and somatosensory feedback. Some speakers may have greater reliance on somatosensory feedback (tongue and jaw) and thus they are not affected by auditory perturbation, whereas others may have greater reliance on auditory feedback and therefore exhibit large compensatory and adaptive responses (Houde and Jordan, 2002; Purcell and Munhall, 2006). In line with this speaker-specific sensory preference, we could argue that a *speaker-specific mode preference* exists (i.e., a tradeoff between the feedback mode and the feedforward mode). If the feedback mode outweighs the feedforward mode, then heavy reliance on auditory feedback could make the individual produce a large proportion of opposing responses (i.e., they would be in the opposer class). If the feedforward mode outweighs the feedback mode, pitch perturbations could be regarded as alien voices and thus shadowing-like following responses would be produced (i.e., they would be in the follower class). The relative weighting of feedback and feedforward modes has also been found in loudness perturbations on the first language (L1) and the second language (L2) production (Cai et al., 2020), where L1 speech production relied more on feedforward control (attenuated Lombard effect) and L2 speech production depended more on feedback control (enhanced Lombard effect).

More importantly, the current research discovered that at the individual level, more than half of speakers had no mode preference, and their opposing responses and following responses alternated across trials (i.e., classified as the switcher class). It seems that stimulus specificity is not an influential factor, because switching patterns were found in both vowel production (the current study and Alemi et al. (2020)) and tone word production (Ning, 2022). Perceptual acuity to pitch may not explain the phenomenon either, because using just-noticeable-difference pitch-shift stimuli did not prevent speakers from exhibiting switching patterns (Alemi et al., 2020). Although we did not examine participants’ perceptual acuity to pitch, it is likely that pitch sensitivity may not play an essential role in determining response inconsistency across trials. We speculate that the switching pattern may have to do with the expectancy of perturbation direction and with the time-scale within an experiment (see the next section for discussion).

### The role of predictability in response strategies

Our three-class latent class regression modeling indicates that switchers were likely to appear when pitch-shift stimulus direction was unpredictable (the random condition; see Figure 3). Opposing responses and following responses alternated when unexpected downward or upward shifts come as a surprise. In other words, the uncertainty of pitch-shift stimulus direction enhances the extent of response inconsistency. However, when pitch-shift stimulus was predictable (the down-only and up-only conditions), the number of followers considerably increased, particularly in the down-only condition. This observation supported our hypothesis and it is consistent with previous studies showing that following responses are more likely to be observed in the predictable condition than they are in the unpredictable condition (Behroozmand et al., 2012; Korzyukov et al., 2012). Following responses derived from the implementation of feedforward mode could be issued efficiently without incorporating feedback information. Therefore, when speakers are pre-informed of the direction of auditory perturbations, the weighting of auditory feedback would be lower and the feedforward route would be recruited.

Overall, we identified three interconnected patterns: (a) Speakers tended to have no speaker-specific mode preference when pitch-shifts are unpredictable (i.e., switchers); (b) the number of followers whose feedforward mode outweighs the feedback mode was conditional on the predictability of pitch-shift stimulus direction; and (c) the number of opposers whose feedback mode outweighs the feedforward mode was unaffected by the expectancy of pitch-shift stimulus direction, maintaining one-third of the population. The last point implies that opposers might be the most rigid group. When the feedback mode is implemented, speakers tend to rely on their auditory feedback no matter whether pitch-shift stimulus direction is predictable (redundant).

Another interesting observation in the latent class regression modeling was that the degree of response consistency was associated with the time-scale (see Figure 3). Our results show that a particular mechanism (opposing or following) was implemented initially, the two mechanisms alternated in the middle phase, and then the pitch-shift response was finalized with a particular mechanism near the end phase. The time-varying changes in response types suggest that response consistency can be achieved within a short block of 30 trials and that sensorimotor learning happens during the test. No matter which response mechanism is implemented, individual participants may eventually learn to deal with environmental (auditory) perturbations within a short time.

### The Behavioral performance in each latent class

The absence of significant differences in response onset time suggests that both opposing and following responses were equally efficient in the sensorimotor integration for all classes of speakers. Regarding the response peak amplitude, the three classes were significantly affected by pitch-shift stimulus direction and response type. When pitch perturbations were predictable (down-only and up-only), we found a directional response pattern for opposers and switchers, but not for followers: Pitch-increasing responses (oppose downshifts or follow upshifts) were significantly *smaller* than pitch-decreasing responses (oppose upshifts or follow downshifts). However, this directional pattern was contradictory to previous findings in Chen et al. (2007), and Liu et al. (2011), and Ning (2022), where an opposite pattern was discovered (pitch-increasing responses larger than pitch-decreasing responses). One potential reason for the inconsistent experimental results could be the predictability of pitch-shift stimulus direction. Whereas the three previous studies examined vocal responses to *unpredictable* pitch-shift stimuli, the current study discovered the directional response pattern in the *predictable* pitch-shift stimulus conditions. Additionally, the nature of the test stimuli (tone words in Ning (2022), English sentence in Chen et al. (2007), and simple vowel in the present study and Liu et al. (2011)) and data analysis techniques (whether opposing and following responses were both considered) may play a role. Further research is required to justify whether the directional response pattern is convincing.

When pitch perturbations were unpredictable (random), opposers’ opposing responses were smaller than switchers’ opposing responses. Large compensation in previous studies has been associated with overreliance on auditory feedback, which can be seen in autistic individuals with 16p11.2 Deletions (Demopoulos et al., 2018), speakers with cerebellum degeneration (Parrell et al., 2017), and L2 learners (Ning et al., 2014, 2015; Cai et al., 2020). This result suggests that though the feedback mode can be implemented in both opposers and switchers, the degree of reliance on auditory feedback was not the same. Switchers, with no preferred response type, tended to put more reliance on auditory feedback than opposers did. However, followers’ following responses were consistently huge, compared to switchers’ following responses. This outcome indicates that the feedforward control, not executed to reduce perceived errors or to increase stabilization, was greatly used by the followers who would largely shadow the vocal pitch. Though the feedforward mode may be implemented from time to time in switchers, the degree of change in their following responses was still less than that of followers. In other words, opposers may correct for the mismatch between perceived pitch and actual pitch without excessively relying on auditory feedback; followers can shadow the perceived pitch *via* using the feedforward control at full strength; and switchers wandering between the two mechanisms would produce medium-sized responses.

We may view the dichotomy of predictability from a different perspective: attentional load. Tumber et al.’s (2014) research on the divided attention examined how speakers responded to pitch perturbation while they had to simultaneously identify target stimuli in a visual stream of letters. They found that in the dual-task (i.e., higher attentional load), less attention was available for monitoring auditory feedback, and thus it led to smaller vocal compensation, compared to the single-task (no letter identification). However, an opposite result—smaller compensation in the low attentional load condition—was found in Liu et al. (2018), where the participants produced sustained vowels while they had to count the number of pitch perturbations and the number of red light flashes they saw on the computer screen. Liu et al. (2018) speculated that the opposite result may be due to the involvement of working memory in counting: vocal compensation would be enhanced when working memory is engaged. Although we did not manipulate divided attention in the present study, the predictable conditions (down-only and up-only) may resemble a case where participants would pay less attention to the expected pitch-shift stimuli, whereas the unpredictable condition may recruit more attention to the pitch-shift stimuli. Our behavioral result—smaller peak amplitudes in the down-only condition than in the random condition—confirms that when more attention is allocated to auditory feedback, enhanced vocal compensation is expected.

Unpredictable perturbations may be regarded as noise or variation that would occur in the process of motor learning. When we learn to drive a car, play with a ball, or speak a new language, we continuously adjust our motion in response to error signals in our feedback systems. The behavioral results from the three latent classes may help us to predict an individual’s capacity to learn new motor skills. It seems that opposers would be the most rigid group where the motor memories would be updated in the slowest way. Followers may begin the update early when noise or variation appears. However, after acquiring robust internal representations for a motoric plan (as an expert would), we eventually have to become opposers so that we can be less affected by unexpected perturbations. The implication from latent class analysis should be used with caution as we only had 30 trials in each condition, far fewer than the number of trials required for motor learning. How speakers may change their response patterns over a longer period time requires further research.

## Conclusion

The present study identified three classes of speakers in terms of their pitch-shift response consistency over 30 trials within an experiment: 57% switchers, 28% opposers, and 15% followers. In other words, more than a half of speakers had no fixed response type. The latent class regression modeling results supported the hypothesis that followers are more likely to appear in the predictable condition than in the unpredictable condition. Closer inspection of the levels of response consistency in different time phases shows that a particular mechanism (opposing or following) was initially implemented, the two mechanisms alternated in the middle phase, and then finally the pitch-shift response was featured as a particular mechanism near the end phase. Furthermore, small opposing responses in opposers and large following responses in followers suggest that the feedback mode and the feedforward mode represent two distinct mechanisms and the effort may be used disproportionally by individuals.

## Data availability statement

The raw data supporting the conclusions of this article will be made available by the authors, without undue reservation.

## Ethics statement

The studies involving human participants were reviewed and approved by Research Ethics Office at National Taiwan Normal University. The patients/participants provided their written informed consent to participate in this study.

## Author contributions

LH contributed to conception and design of the study, performed the statistical analysis, and wrote the entire manuscript.

## Funding

This project is funded by Ministry of Science and Technology, Taiwan (Grant No. MOST 110-2410-H-003-010-MY2).

## Conflict of interest

The author declares that the research was conducted in the absence of any commercial or financial relationships that could be construed as a potential conflict of interest.

## Publisher’s note

All claims expressed in this article are solely those of the authors and do not necessarily represent those of their affiliated organizations, or those of the publisher, the editors and the reviewers. Any product that may be evaluated in this article, or claim that may be made by its manufacturer, is not guaranteed or endorsed by the publisher.
